# The Development of an Instagram Reel-Based Bystander Intervention Message Among College Students: Formative Survey and Mixed Methods Pilot Study

**DOI:** 10.2196/66769

**Published:** 2025-01-27

**Authors:** Leticia Couto

**Affiliations:** 1 College of Communication, DePaul University, Daley Bldg, 14 E Jackson Blvd, Chicago, IL, 60605, United States, 1 (312) 362-8600

**Keywords:** bystander intervention, message development, sexual health, college, student, sexual violence, bystander, reel-based, Instagram, social media, short message, formative research, mixed methods, social norms, perceived behavior, qualitative, behavioral health, digital health

## Abstract

**Background:**

Bystander intervention is a common method to address the ubiquitous issue that is sexual violence across college campuses. Short messages that incentivize bystander intervention behavior can be another tool to fight sexual violence.

**Objective:**

This study aimed to conduct formative research surrounding social norms and bystander barriers to pilot and develop Instagram (Meta) reel-based messages addressing bystander intervention among college students.

**Methods:**

The first step was to conduct a formative survey to identify peer norms and actual behavior of the intended population. Once that data were collected, a mixed methods message pilot was conducted by a survey where participants randomly saw 5 of the 12 messages developed, assessing them for credibility, perceived message effect, and intended audience.

**Results:**

The formative survey was conducted among 195 college students from the same institution, and the pilot test was conducted among 107 college students. The formative survey indicated a discrepancy between perceived peer behavior and actual behavior of the participants in all 3 measures, allowing for the development of normative messaging. The pilot testing indicated the credibility was acceptable (eg, mean 3.94, SD 1.15 on a 5-point scale) as well as the perceived message effect (eg, mean 4.26, SD 0.94 on a 5-point scale). Intended audiences were also identified and reached. Qualitative results indicated that the messages may have lacked credibility, although the quantitative results suggest otherwise.

**Conclusions:**

Participants understood the messages concerned bystander intervention, and perceived message effects results indicated the messages to be effective in assisting bystander intervention engagement by normative messaging. Messages were considered credible and reached the intended audience. The qualitative results provided further insights on how the messages can be adapted before being tested for effects. Future research should focus on further adapting the messages and testing their effects among the studied population.

## Introduction

### Background

Bystander intervention trainings and messages are two of the main ways universities in the United States attempt to address the sexual violence crisis in college campuses [1,2]. Although it is common for bystander intervention messaging (here, *bystander intervention* is defined as the ability to identify situations in which a third party may assist someone in a risky and act to intervene [3]) to use bystander models as a theoretical framework [4,5], the social norms approach is also used to address sexual violence interventions [6]. For the social norms approach to be successful, it is necessary that the population of the message experiences pluralistic ignorance, where there is a difference between their own behavior and their estimate of peer behavior [6]. The social norms approach suggests that people who believe that their peers are engaging in a behavior are more likely to engage in that behavior themselves [6], whereas bystander models, in particular bystander barriers, suggest that the belief that others will take responsibility removes their own responsibility to engage in bystander intervention [7]. Many trainings and programs exist that focus on stimulating bystander intervention behavior [8,9], but less is known about the role of supporting media messages. In addition, it is necessary to investigate how these 2 opposing perspectives may be perceived as messaging mechanisms for college students.

### Sexual Violence and Bystander Intervention

There is a sexual assault crisis on college campuses. Estimates indicate that approximately 1 in 4 college women are sexually assaulted during their time in college [1], which is high compared with the rate of 1 in 5 among the general population of women in the United States [2]. In addition, certain populations seem to be more at risk, such as first-year students [10] and LGBTQ+ (lesbian, gay, bisexual, transgender, queer+) students [1]. Universities across the country have tried to curb the problem by using a series of prevention efforts against sexual violence, with one of the most popular ones being bystander intervention [11].

Bystander intervention training and campaigns, including in-person and online training, are effective ways of increasing intervention intentions among college students in multiple scenarios [5,12]. An example of these campaigns is Green Dot, which features a focus on motivational speech and in-person training [12]. Over 500 universities in the United States have adopted Green Dot [13]. It is worth noting, however, that the bystander effect has been brought into question [14,15]. This can be in part due to its questionable origins in the Kitty Genovese issue [16]. Even still, this event led to an eventual refinement of what it means to intervene as a bystander, and the trainings developed from there proved to be successful [4,5,12,17,18].

### Instagram as a Channel for Interventions

Using Instagram as a platform for public health concerns has been studied mostly in an exploratory manner [19]. However, some studies suggest that the issue is not necessarily reach but the ability of the entities governing these accounts to communicate with their publics [20]. In addition, such messaging developed for Instagram can be easily adapted for other channels, facilitating eventual scalability efforts.

Instagram reels, in particular, are more popular than regular Instagram posts and stories because they have better reach and can be found more easily than regular posts [21]. They also have more editing features that can be used to make messages engaging for the audience [21]. Instagram is also highly used among college-aged populations, more so than other platforms [22], such as Twitter (now X), Facebook, and Snapchat. Thus, the Instagram reel-inspired format can be an optimal channel for short and targeted messaging.

### This Study

In this study, a formative survey was conducted to assess if pluralistic ignorance exists in a college population in relation to bystander intervention behavior in sexual assault situations. This step needed to be established first to ensure a social norms–based message would be feasible. In the presence of pluralistic ignorance, 3 theoretically based Instagram reel-based video messages with short scripts messages were developed and piloted in terms of credibility, intended audience, perceived message effectiveness, and a manipulation check of the conditions. One condition on bystander behavior barriers, focusing on showing participants only the actual behavioral data reported in the survey, was called the message readjustment condition in this study; another condition was based on the social norms approach, which was called the norm reinforcement condition in this study, as it focused on showing participants only the normative information from the data collected in the survey; and 1 combined both theoretical frameworks, showing both the normative information and the behavioral information with the intent of highlighting the discrepancy between the 2. The goal of this study is to assess the perceived message effectiveness, credibility, and intended audience reach of these messages pertaining to bystander intervention.

## Methods

### Formative Survey

In the first semester of 2023 in a large, public, American university, data were collected from 195 undergraduate students aged 18 to 29 years (mean 20.52, SD 2.22 years), most of whom identified as women (118/195, 60.5%), cisgender (184/195, 94.4%), heterosexual (123/195, 63.1%), White (139/195, 71.6%), not Hispanic (110/195, 56.7%), and middle class (124/195, 63.6%). Participants were recruited from a randomized list of 2000 emails from the university’s registrar’s office, representative of the university as whole.

### Formative Research Measurements

#### Bystander Intervention Behavior

Three items were adapted from Hust et al [23]. Participants were asked to indicate how much they agree (5) or disagree (1) with statements related to perceived norms. Specifically, these items concerned the participants own behavior. These items included “I have made sure my friends are ok if I see him/her in an uncomfortable sexual situation at a party” and “I have discouraged a friend who said they planned to get someone drunk to have sex.” The Cronbach α was assessed for the entire sample, for a value of .83.

#### Bystander Intervention Perceived Norms

Three items were adapted from Hust et al [23]. Participants were asked to indicate how much they agree (5) or disagree (1) with statements related to perceived norms. Specifically, these items refer to how they perceived their peers would act in these situations. Items include the stem “most of my friends would,” followed by items such as “make sure their friend is ok if they see him/her in an uncomfortable sexual situation at a party” and “discourage a friend who said they planned to get someone drunk to have sex.” The Cronbach α was assessed for the entire sample, for a value of .8.

### Formative Research Results

Table 1 demonstrates that there was a discrepancy between peer perception and actual behavior of participants. The means were higher among the normative statements (bottom 3) as compared with their actual behavior items counterparts, indicating that participants said they more strongly agreed with the normative statements. This indicates that there was an opportunity for a social norms message to be developed, as pluralistic ignorance was identified.

**Table 1.** Means and SDs of items used for message development.

Items	Mean (SD)	n/N*^a^* (%)
I have made sure my friends are ok if I see him/her in an uncomfortable sexual situation at a party.	4.43 (1.08)	163/195 (83.6)
I have warned someone if I saw a drug being slipped into their drink.	3.63 (1.41)	93/192 (47.7)
I have discouraged a friend who said they planned to get someone drunk to have sex.	3.73 (1.43)	107/193 (55.5)
Most of my friends would make sure their friend is ok if they see him/her in an uncomfortable sexual situation at a party.	4.86 (0.41)	191/195 (98)
Most of my friends would warn someone if they saw a drug being slipped into their drink.	4.89 (0.38)	191/195 (98)
Most of my friends would discourage a friend who said they planned to get someone drunk to have sex.	4.82 (0.47)	188/195 (96.4)

a Percentage that indicated either “agree” (5) or “somewhat agree” (4) for each of these statements.

### Pilot Testing

Messages were developed based on the items tested in the formative research survey step. A total of 12 messages were developed, 3 per condition. The experimental conditions and the theoretical basis for each can be found on Table 2.

**Table 2.** Experiment conditions based on theoretical frameworks.

Condition	Theoretical framework	Outcomes
Norm readjustment	Bystander barriers [7]	Intention to take responsibility
Norm reinforcement	Social norms approach [6]	Perceived peer norm
Norm readjustment and reinforcement	Social norms approach and bystander barriers [6,7]	Intentions
Control	—^a^	—

a Not applicable.

Data were collected in October of 2023. This data collection was exempt from institutional review board (IRB) review for similar reasons as the formative survey, where the university’s IRB reviewed the proposal and indicated that a full-board review was not necessary considering the minimal risks. To be eligible to participate, participants had to be currently in the United States, be an undergraduate student at the university being studied, and be between the ages of 18 and 29 years. Participants were recruited through a pool of students taking communication courses and were compensated with extra credit. Other recruitment methods were attempted, such as social media recruitment, but they yielded very few participants. These participants were compensated with Starbucks gift cards. These 3 conditions were added as screeners to ensure only participants who conformed to them participated in the pilot. This type of pilot testing strengthens the validity and reliability of the intervention, and it has been used in similar efforts [24,25].

Participants saw 5 of 12 Instagram reel-based short video messages developed in random order (3 per condition: norm readjustment, norm reinforcement, combined condition, and control). The following instructions were provided to the participants before they started the pilot: “Next, you will see 5 short videos. You will answer some questions about those videos and then some overall questions. Please make sure you can hear the videos as you watch them. Please click on the button on the bottom right to continue.” Participants were also provided with the following description of bystander intervention: “Definition of bystander intervention: Bystander Intervention is recognizing a potentially risky situation or interaction and choosing to respond in a way that could positively affect the outcome. For the purposes of this study, we are mostly referring to intervening in situations that could escalate to sexual violence.” As these messages had to be highly tailored, the messages referred to the students with the name of the university’s mascot (eg, if the university’s mascot was a tiger, the participants were called tigers). These references were removed from this paper to ensure the anonymity of the participants and an unbiased peer review process.

A total of 107 individuals participated in the pilot, but 81 participants responded to demographics items. Participants’ ages ranged from 18 to 24 years (mean 20.16, SD 1.33 years). Most identified as women (52/81, 64.2%) and cisgender (80/81, 98.8%). Most identified as heterosexual (60/81, 74.1%), but a considerable portion of the sample either chose a sexual minority (15/81, 18.4%) or chose not to answer (6/81, 7.4%). Most participants were non-Hispanic/Latine (44/81, 54.3%) and were Caucasian/White (61/81, 75.3%). Most participants also self-identified as middle class (58, 71.6%).

### Pilot Measures

#### Perceived Message Effectiveness

An adaptation of the 3 items from the University of North Carolina’s Perceived Message Effectiveness Scale (UNC PMES) was used to measure perceived message effectiveness [26]. Example items include “This message makes me concerned about the lack of bystander intervention engagement on campus” and “This message discourages me from not engaging in bystander intervention.” Participants were asked to indicate how much they agree (5) or disagree (1) with these statements. The Cronbach α for the messages varied between .68-.92. The average α was .87.

#### Credibility

To measure credibility, participants were asked how much they agreed (5) or disagreed (1) that the message was trustworthy, credible, believable, and realistic. The 4 items were combined to form a credibility scale. This scale was self-developed based on previous literature exploring credibility of messages [27]. The Cronbach α range for the credibility composite scores was .89-.95 with an average of .92.

#### Intended Audience for the Message

Participants were asked to indicate how much they disagree (1) or agree (5) that the following groups are the intended audience of the message: college students, people between 18‐29 years old, and [university] students. The goal of this item was to assess whether the message resonates with its intended audience (ie, college students, [university] students, people aged between 18‐29 years). These items were self-developed to address the specific intended audiences of these messages.

#### Manipulation Checks

A total of 6 manipulation checks were developed. The participants were asked to indicate how much they agreed (5) or disagreed (1) with the statements that had the stem “the message I saw,” followed by “encouraged me to be an active bystander,” “encouraged me to be part of the group that helps” (items that distinguished control messages from the other messages), “suggests very few [university mascot] help others at parties” (refers to readjustment condition messages and combination condition messages), “suggests a lot of [university mascot] help others at parties” (refers to reinforcement condition), “suggests [university mascot] trust their friends” (refers to reinforcement condition and combination condition messages), and “contains tips on how to party safely” (refers to the control messages). These were evaluated based on means and how responses compared to one another.

#### Open-Ended Items

Participants were asked to indicate what they liked and disliked about the messages, as well as to identify what they believed to be the purpose of the message.

### Initial Message Design

**Figure 1.**
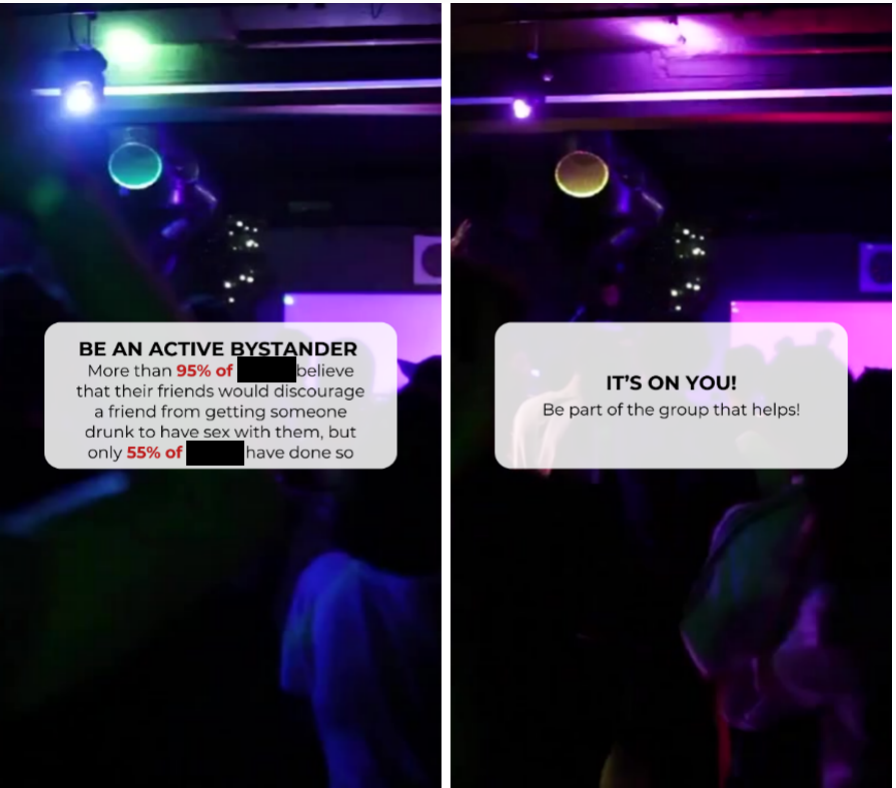
Piloted message example.

The messages were designed on Canva, using available music and visuals. The script was created based on the bystander items described in this paper. The scripts are approximately 10‐15 words from each other in terms of length. Please refer to Textbox 1 for a complete breakdown of each initial message, the condition that it belongs to, and the number by which the message is referred to in the *Results* section. Figure 1 shows an example of the message.

Textbox 1. **Initial messages and the conditions they belong to (message numbers are those referred to in the *Results* section).**

**Norm readjustment**

Message 1:

*BE AN ACTIVE BYSTANDER*

*Only 48% of [UNIVERSITY MASCOT] have told someone that their drink had been drugged*

*IT’S ON YOU!*

*Be part of the group that helps!*

­

Message 2:

*BE AN ACTIVE BYSTANDER*

*Only 84% of [UNIVERSITY MASCOT] have made sure their friend was ok if they seem to be in an uncomfortable situation at a party*

*IT’S ON YOU!*

*Be part of the group that helps!*

­

Message 3:

*BE AN ACTIVE BYSTANDER*

*Only 55% of [UNIVERSITY MASCOT] have discouraged a friend from getting someone drunk to have sex with them*

*IT’S ON YOU!*

*Be part of the group that helps!*

­

**Norm reinforcement**

Message 4:

*BE AN ACTIVE BYSTANDER*

*More than 97% of [UNIVERSITY MASCOT] believe their friends would tell someone that their drink had been drugged*

*IT’S ON YOU!*

*Be part of the group that helps!*

­

Message 5:

*BE AN ACTIVE BYSTANDER*

*More than 97% of [UNIVERSITY MASCOT] believe that their friends would make sure another friend was ok if they seem to be in an uncomfortable situation at a party*

*IT’S ON YOU!*

*Be part of the group that helps!*

­

Message 6:

*BE AN ACTIVE BYSTANDER*

*More than 95% of [UNIVERSITY MASCOT] believe that their friends would discourage a friend from getting someone drunk to have sex with them*

*IT’S ON YOU!*

*Be part of the group that helps!*

­

**Norm readjustment and reinforcement**

Message 7:

*BE AN ACTIVE BYSTANDER*

*More than 97% of [UNIVERSITY MASCOT] believe their friends would tell someone that their drink had been drugged, but only 48% of [UNIVERSITY MASCOT] have done so*

*IT’S ON YOU!*

*Be part of the group that helps!*

­

Message 8:

*BE AN ACTIVE BYSTANDER*

*More than 97% of [UNIVERSITY MASCOT] believe that their friends would make sure another friend is ok if they seem to be in an uncomfortable situation at a party, but only 84% of [UNIVERSITY MASCOT] have done so*

*IT’S ON YOU!*

*Be part of the group that helps!*

­

Message 9:

*BE AN ACTIVE BYSTANDER*

*More than 95% of [UNIVERSITY MASCOT] believe that their friends would discourage a friend from getting someone drunk to have sex with them, but only 55% of [UNIVERSITY MASCOT] have done so*

*IT’S ON YOU!*

*Be part of the group that helps!*

­

**Control**

Message 10:

*BE SAFE AT YOUR NEXT PARTY!*

*At parties, make sure you know where the nearest exit is in case of an emergency. You never know when you might have to bounce!*

*PLAN AHEAD!*

*Safe partying is more fun!*

­

Message 11:

*BE SAFE AT YOUR NEXT PARTY!*

*At parties, make sure you know where you place important belongings like wallet and keys. You never know when you may need to bounce!*

*PLAN AHEAD!*

*Safe partying is more fun!*

­

Message 12:

*BE SAFE AT YOUR NEXT PARTY!*

*At parties, make sure you know how you are getting back home. You never know when you may need to bounce!*

*PLAN AHEAD!*

*Safe partying is more fun!*

### Qualitative Analysis

To evaluate the qualitative open-ended questions, thematic analysis was used [28,29]. Thematic analysis was chosen instead of other potential qualitative methods to allow for a more organic identification of themes and discussion among coders. The coders were the author and a colleague in the same discipline as the author. The first step in Braun and Clarke’s [28,29] thematic analysis is to become familiarized with the data. The coders read all the responses before starting to code them. After that, the responses were prepped in a spreadsheet format. The coders highlighted cells based on how they may represent a theme. If a segment may be referring to 2 or more themes, coders were to add a column next to the column where the segments were and highlight the cell next to the original segment. This procedure repeated itself for each question (likes, dislikes, and purpose) and per message (9 messages total). Although 12 messages were tested, the qualitative coding only happened for the messages that were related to bystander intervention and not the control messages (messages 10‐12). Thus, there is a total of 24 units to analyze, where each has between 28‐37 segments as responses. A total of 930 segments were coded. Then, we used 1 set of messages to train, where we coded the qualitative responses related to message 1 while we were together, but the coding happened separately. During the training, we established the scope of the themes, and we established that for the priming question, we were coding for 2 themes: participants understood the message, and participants did not understand the message. After coding the segments associated with message 1, we found that we agreed in most instances on the development of themes and how we approached the priming segments. We used the segments associated with messages 1‐4 to establish the most common themes and used those themes to code the rest of the segments in messages 5‐9. Thus, approximately 44% of the data were used to establish the themes, and those themes were reinforced using the rest of the data. We then moved on to code the segments associated with messages 2‐9 by ourselves, and compared the coding of the coder and my coding to assess if there was a need for us to meet again and discuss any major disagreements. Minor disagreements were addressed by email, phone calls, and text messages.

### Statistical Analysis

The pilot was assessed using descriptive statistics only, as the goal of this stage of the messages is to check for credibility, perceived audience, manipulation checks, and perceived effects. Means and frequencies were used to make these assessments.

### Ethical Considerations

Participants were compensated with the opportunity to enter a drawing to receive a gift card by Tango Cards in the value of US$100 (three available cards), US$50 (six available cards), or US$25 (eight available cards) in the formative survey and with extra credit in a communication course of their choice for their participation in the pilot for most participants, with very few receiving Starbucks gift cards instead. Data collection happened through an online survey and was collected anonymously. The drawing information was completely unlinked from the survey responses. This study was exempt from review by the author’s institution. Exempt review means the protocol was reviewed by a member of the university’s IRB, but a full-board review was not deemed necessary, as risks were considered minimal.

## Results

### Qualitative Results

#### Eye-Catching Statistics and Information

In general, participants indicated that they appreciated how informative the messages were and that statistics were provided. One participant wrote that they liked that “[The message] gives a statistic that catches your attention,” and another participant mentioned, “I like the percentages displayed in the video.” This was a pattern across all test messages where these were things participants appreciated in the messages.

#### Production Passed the Vibe Check

What is conceptualized here as production value is both aspects, such as the video chosen and the music in the background, as well as descriptions of “vibes” and entertainment value. Participants indicated that the message was “upbeat” and that they liked the background music often. They also appreciated how short the message was while still providing valuable information. One participant said that “[The message] was simple and effective,” and a different participant said that “the background video and music was engaging.”

#### No Source to Back It Up? Participants Disliked the Lack of Source for the Information Provided

The most common theme of dislike toward the message was the lack of source for the statistics provided. Multiple participants expressed disdain or skepticism toward the statistics, which could come from a lack of source but also a reluctance to accept that they were true in relation to fellow college students in their institution. One participant said that they disliked that there was “no source to back it up,” while another one explained that they disliked that the message “did not show how they got the statistic.”

#### Maybe a Bit Boring

This theme was developed from participants mentioning that the messaging is “boring” or “not very engaging.” This theme is hard to conciliate with the presence of participants who did express they appreciated that the message was short and kept them interested throughout. One participant said that “[The message] is boring and unengaging,” and another indicated that “[The message] was boring to watch.”

#### Message Understood!

In general, participants understood what the messages were trying to achieve with their call to action and information. One participant explained that their understanding of the message was that “this message is telling people that [UNIVERSITY MASCOT] intervene when it comes to getting people drunk for sex, and so should they,” while another participant indicated that “Be an active bystander” was the main message. This is important information to be taken together with the results from the quantitative analysis described in the following paragraphs.

### Quantitative Results

#### Perceived Message Effectiveness

To assess the quantitative results, the mean of the scales was used in reference to the midpoint of the scale, that is, above the midpoint represented acceptance and below midpoint represented rejection. All bystander-related messages (1-9) were above the midpoint (3 on a 1‐5 scale, where 1=disagree and 5=agree) in terms of perceived message effectiveness. The bottom 2 means in perceived message effectiveness belonged to 2 control messages: message 10 (mean 2.77, SD 1.36) and message 12 (mean 2.85, SD 1.12). Although all condition messages (1-9) were above the midpoint, the lowest mean among them belonged to message 5 (mean 3.19, SD 1.33), and that was lower than the mean for message 11 (mean 3.26, SD 1.27), which is a control message. The highest means belonged to message 6 (mean 3.94, SD 1.15) and message 9 (mean 3.85, SD 1.18). The results can be seen in their completion in Table 3.

**Table 3.** Perceived message effectiveness results.

Condition and message		Number of participants per condition	Mean (SD)	
**Readjustment**				
	1	37	3.46 (1.2)	
	2	38	3.56 (1.24)	
	3	38	3.76 (1.12)	
**Reinforcement**				
	4	34	3.79 (1.13)	
	5	36	3.19 (1.33)	
	6	36	3.94 (1.15)	
**Combination**				
	7	36	3.67 (1.29)	
	8	37	3.69 (1.24)	
	9	35	3.85 (1.18)	
**Control**				
	10	40	2.77 (1.36)	
	11	35	3.26 (1.27)	
	12	36	2.85 (1.12)	

#### Credibility

When assessing the means for credibility for each message, we see that generally, all messages were above the midpoint (3 on a 1‐5 scale). The highest was achieved by message 6, at mean 4.26 (SD 0.94), and the lowest were obtained for messages 5 and 11, at mean 3.90 (SD_5_ 0.96, SD_11_ 0.98). The mean (SD) values are presented in Table 4.

**Table 4.** Message credibility results.

Condition and message		Number, n	Mean (SD)	
**Readjustment**				
	1	36	4.20 (0.74)	
	2	38	3.95 (0.87)	
	3	38	3.82 (1.03)	
**Reinforcement**				
	4	34	4.30 (0.87)	
	5	36	3.90 (0.96)	
	6	36	4.26 (0.94)	
**Combination**				
	7	36	4.22 (0.94)	
	8	37	4.18 (0.79)	
	9	35	4.10 (0.97)	
**Control**				
	10	40	4.06 (0.75)	
	11	34	3.90 (0.98)	
	12	36	4.11 (1)	

#### Message Audiences

Participants were asked to assess what audiences they believed the messages they saw would be most suited to. That was done using a Likert-type scale ranging from 1 (disagree) to 5 (agree). All major intended audiences for the message, college students, [university] students, and people who are 18‐29 years old, had high means in terms of participants perceiving the messages as being appropriate for them as an audience. Among the condition messages, the highest one was Message 7 among college students (mean 4.86, SD 0.42), and the lowest one was Message 3 among people between ages 18‐29 (mean 4.37, SD 1.08). Table 5 shows a full breakdown of results.

**Table 5.** Audience perception means and SDs.

Audience, condition, and message			Number, n	Mean (SD)	
**College students**					
	**Readjustment**				
		1	36	4.78 (0.54)	
		2	38	4.58 (0.79)	
		3	38	4.68 (0.7)	
	**Reinforcement**				
		4	34	4.79 (0.54)	
		5	36	4.67 (0.63)	
		6	35	4.77 (0.55)	
	**Combination**				
		7	36	4.86 (0.42)	
		8	37	4.73 (0.65)	
		9	35	4.77 (0.49)	
	**Control**				
		10	40	4.67 (0.66)	
		11	34	4.62 (0.74)	
		12	36	4.78 (0.59)	
**People aged between 18‐29 years**					
	**Readjustment**				
		1	36	4.47 (1.06)	
		2	38	4.53 (0.73)	
		3	38	4.37 (1.08)	
	**Reinforcement**				
		4	34	4.56 (0.93)	
		5	36	4.44 (0.91)	
		6	35	4.6 (0.74)	
	**Combination**				
		7	36	4.61 (0.87)	
		8	37	4.57 (0.77)	
		9	35	4.57 (0.95)	
	**Control**				
		10	40	4.6 (0.78)	
		11	34	4.41 (0.86)	
		12	36	4.81 (0.4)	
**[University name] students**					
	**Readjustment**				
		1	36	4.67 (0.76)	
		2	38	4.61 (0.76)	
		3	38	4.68 (0.7)	
	**Reinforcement**				
		4	34	4.74 (0.71)	
		5	36	4.81 (0.47)	
		6	35	4.74 (0.7)	
	**Combination**				
		7	36	4.81 (0.53)	
		8	37	4.73 (0.65)	
		9	35	4.74 (0.61)	
	**Control**				
		10	40	4.65 (0.62)	
		11	34	4.38 (0.99)	
		12	36	4.67 (0.72)	

#### Manipulation Checks

The goal of the manipulation checks was to ascertain if the message conditions were properly different from each other in what they were trying to achieve. A total of 6 statements were developed to assess different aspects of the messages and check that manipulation efforts are working. Results indicated that, indeed, all message conditions were properly differentiated from each other. For example, the control condition message 11, which had the highest mean among the control messages, had a lower mean in the manipulation check “the message I saw encouraged me to be an active bystander” (mean 3.15, SD 1.46) in a 5-point scale than the lowest noncontrol message (message 3: mean 3.74, SD 1.35). Full results can be found in the Table 6.

**Table 6.** Manipulation check descriptive statistics.

Manipulation check, condition, and message			Number, n	Mean (SD)	
**The message I saw encouraged me to be an active bystander**					
	**Readjustment**				
		1	36	4 (1.24)	
		2	38	3.82 (1.18)	
		3	38	3.74 (1.35)	
	**Reinforcement**				
		4	34	4.18 (1.24)	
		5	35	4.03 (1.2)	
		6	35	4.06 (1.35)	
	**Combination**				
		7	36	4.17 (1.23)	
		8	37	4.16 (1.07)	
		9	34	4.09 (1.26)	
	**Control**				
		10	39	2.97 (1.58)	
		11	34	3.15 (1.46)	
		12	36	2.86 (1.48)	
**The message I saw encouraged me to be part of the group that helps**					
	**Readjustment**				
		1	36	4.5 (0.74)	
		2	38	4.08 (1)	
		3	38	4.13 (0.99)	
	**Reinforcement**				
		4	34	4.41 (0.96)	
		5	35	4.37 (0.81)	
		6	35	4.63 (0.81)	
	**Combination**				
		7	36	4.47 (0.94)	
		8	37	4.49 (0.77)	
		9	34	4.47 (0.75)	
	**Control**				
		10	39	3.05 (1.62)	
		11	34	3.65 (1.28)	
		12	36	3.17 (1.52)	
**The message I saw suggests very few [university mascot] help others at parties**					
	**Readjustment**				
		1	36	3.94 (1.04)	
		2	38	2.95 (1.16)	
		3	38	3.84 (1.24)	
	**Reinforcement**				
		4	34	3.06 (1.67)	
		5	35	3.03 (1.67)	
		6	35	2.8 (1.71)	
	**Combination**				
		7	36	3.97 (1.21)	
		8	37	3.38 (1.34)	
		9	34	3.53 (1.24)	
	**Control**				
		10	39	2.49 (1.3)	
		11	34	2.68 (1.36)	
		12	36	2.5 (1.4)	
**The message I saw suggests a lot of [university mascot] help others at parties**					
	**Readjustment**				
		1	36	3 (1.24)	
		2	38	3.87 (0.84)	
		3	38	2.55 (1.13)	
	**Reinforcement**				
		4	34	3.97 (1.31)	
		5	35	4.23 (1.14)	
		6	35	4.49 (0.7)	
	**Combination**				
		7	36	2.89 (1.47)	
		8	37	3.73 (1.17)	
		9	34	3.06 (1.25)	
	**Control**				
		10	39	2.59 (1.23)	
		11	34	2.97 (1.34)	
		12	36	2.67 (1.29)	
**The message I saw suggests [university mascot] trust their friends**					
	**Readjustment**				
		1	36	3.11 (1.17)	
		2	38	3.74 (0.86)	
		3	38	2.89 (1.23)	
	**Reinforcement**				
		4	34	4.32 (0.95)	
		5	35	4.37 (0.94)	
		6	35	4.43 (0.85)	
	**Combination**				
		7	36	3.44 (1.3)	
		8	37	4.11 (0.84)	
		9	34	3.74 (1.11)	
	**Control**				
		10	39	2.69 (1.22)	
		11	34	3.41 (1.33)	
		12	36	2.83 (1.28)	
**The message I saw contains tips on how to party safely**					
	**Readjustment**				
		1	36	3.22 (1.36)	
		2	38	3.11 (1.43)	
		3	38	2.61 (1.37)	
	**Reinforcement**				
		4	34	3.12 (1.45)	
		5	35	3.2 (1.28)	
		6	35	3.83 (1.29)	
	**Combination**				
		7	36	3.06 (1.55)	
		8	37	3.19 (1.43)	
		9	34	3.15 (1.44)	
	**Control**				
		10	39	4.31 (1.22)	
		11	34	4.35 (0.98)	
		12	36	4.44 (0.84	

## Discussion

### Principal Findings

In this study, the feasibility of a social norms message was assessed by the means of a formative survey study, demonstrating that there was indeed a discrepancy in terms of pluralistic ignorance. The presence of pluralistic ignorance allows for the development of social normative messaging. After establishing the presence of pluralistic ignorance, a message was piloted, and results indicated that the messages reached the intended audience, had the intended perceived effect, were perceived as credible, and the manipulation checks indicated the conditions were properly differentiated from each other while representing their purpose. The feasibility of messages focusing on bystander barriers and combining bystander barriers and the social norms approach was also explored. These messages were also highly accepted among the participants, as well as highly understood. Further research is necessary to explore which theoretical approach is ideal to reach this population in terms of the topic of bystander intervention in sexual assault situations. This was the first study to consider combining opposing approaches to bystander intervention messaging—one where it is suggested that people should consider the behavior of their peers [6] and one where they should take more responsibility for their own actions [7].

Although all 3 approaches showed appropriate levels of perceived message effect and credibility, it is necessary to underscore that the reinforcement condition and the combination condition were particularly successful. This suggests that the bystander barrier associated with taking responsibility may not be as strong of a factor in bystander intervention messaging as it is an attitude to be changed by messaging. The fact that the common denominator among the more successful messages is the presence of normative information is in line with the current literature surrounding sexual violence prevention in the form of media messages [30] as well as theoretical frameworks used to address sexual violence prevention [6,7], further building on it by adding a component that goes beyond norms only. However, more must be explored in terms of combined message by proper experimental designs.

The discrepancy between the qualitative and quantitative results of the pilot in terms of credibility highlighted the need to conduct mixed methods pilots to holistically assess messages that concern public health. Although the quantitative results indicated that credibility was appropriate as is, the qualitative data indicated that there were ways to improve credibility even more. The message should be adjusted before further evaluation of its effects. Possible reasons for this discrepancy could be that, although participants find the message credible overall, as in, the message had face validity to them, they would expect proper sourcing of this information. When asked quantitatively if the messages were credible, they may tend to say it is credible, but when they have the space to expand on that, they explain how this credibility could become more formal.

In terms of practical implications, this study shows the possibility for universities and other organizations that conduct bystander intervention training to provide their publics with messages that can underscore these trainings. It is unlikely that an Instagram reel-based message alone can change one’s behavior. However, media messages can serve to reinforce adopted norms [30-32]. This can, in turn, maintain a change in behavior from a more complex intervention that goes beyond media exposure.

### Limitations

This study, although novel, is not without limitations. The messages were highly tailored for the students of the university where the study was conducted. Although this is ideal for social normative messaging, it can interfere with the scalability of the study. Furthermore, more assessments using inferential statistics are necessary to establish the potential effects of these messages. These are the next steps in this line of research. In addition, the messages lacked proper sourcing, which participants identified particularly in the qualitative results as a flaw. Future developments of these messages should provide proper sourcing to enhance the credibility of this intervention.

### Conclusion

Overall, this study explores how different theories can contribute in different ways to the same public health concern. The messages assessed here have proven to be adequate for the next stage of evaluation with an experimental design. However, this next step would not be as rigorous if the procedures discussed here had not been conducted first.

## Supplementary material

10.2196/66769 Checklist 1 CHERRIES (Checklist for Reporting Results of Internet E-Surveys) checklist of items that apply to this study.

## Abbreviations

IRB: institutional review board
LGBTQ+: lesbian, gay, bisexual, transgender, queer+
UNC PMES: University of North Carolina’s Perceived Message Effectiveness Scale
