# Supplementary material for: The Development of an Instagram Reel-Based Bystander Intervention Message Among College Students: Formative Survey and Mixed Methods Pilot Study
Source: JMIR Form Res. 2025 Jan 27;9:e66769. doi: 10.2196/66769 (PMC11789691; doi:10.2196/66769)
Supplement: Checklist 1 [file formative-v9-e66769-s001.pdf]

CHERRIES checklist

Incentives	Study 1 – Gift card drawing for their vendor of choice, 3 $100, 6 $50, and 8 $25.<br>Study 2 – Gift card to participants that used a snowball sample (very few), extra credit for the majority of participants.
Time/Date	Study 1 – First semester 2023<br>Study 2 – October 2023
Number of Items	Study 1 – 193<br>Study 2 – 276
Review step	Participants were not allowed to go review their answers once submitted.
Unique site visitor	Study 1 – Individual links per email sent, but anonymous.<br>Study 2 – Anonymized completely.
Participation rate	These two pages were the same for both studies. As in, the first page the participants saw was the consent page.
Handling of incomplete questionnaires	Missing data was analyzed and they were both MCAR.
Questionnaires submitted with an atypical timestamp	Removed from dataset.
